# Role of Magnetic Resonance Imaging in Morphometric Alterations of Corpus Callosum in Stroke Patients

**DOI:** 10.7759/cureus.35332

**Published:** 2023-02-22

**Authors:** Buchipudi Sandeep Reddy, Deepti Naik, Anil K Sakalecha, Yashas Ullas L, Kalathuru Uhasai, Jayendra Mannan V

**Affiliations:** 1 Radio-Diagnosis, Sri Devaraj Urs Academy of Higher Education and Research, Kolar, IND; 2 Radio-Diagnosis, Sri Devaraj Urs Medical College, Kolar, IND

**Keywords:** morphometry, mri, gender, corpus callosum, stroke

## Abstract

Background: Corpus callosum plays a role in interhemispheric integration, language, intelligence, and creativity of individuals, hence variations in corpus callosum size are seen in various neurological diseases such as Alzheimer’s and bipolar affective disorder. While the dimensions differ based on gender, age, and ethnicity, pathological variations are seen with some diseases such as vascular dementia, leukoaraiosis, stroke, and carotid artery stenosis. This study was conducted to compare the morphometric alterations of the corpus callosum between normal subjects and stroke patients using magnetic resonance imaging (MRI).

Methods: This was a case-control study conducted on 84 subjects divided into cases and control groups. The widths of the genu, body & splenium, and anterior-posterior (AP) diameter of the corpus callosum were measured and the values were compared among the two groups. Student’s t-test and regression analysis were utilized for the analysis of data and p<0.05 was considered statistically significant.

Results: Sixteen patients (19.04%) belonged to the age range of 18-40 years, 32 (38.09%) belonged to the age range of 41-60 years and 36 (42.8%) belonged to the age group of >60 years. There was no discrepancy between cases and controls or between the age groups. The mean width of genu, body & splenium, and AP diameter was compared between normal individuals and stroke patients. It was noted to be significantly lesser in cases than in controls. The morphometric indices i.e., width of genu, body & splenium, and AP diameter of the corpus callosum in cases versus controls were noted to be 9.8 ± 1.2 vs. 10.27 ± 0.3 mm, p=0.12; 5.1±0.9 vs. 5.3±0.24 mm, p=0.25; 12.11 ± 9.65 vs. 12.52 ± 13.9 mm, p=0.04 (significant) and 71.22±3.1 vs. 72.32±1.2, p=0.23, respectively.

Conclusion: This study showed that patients with stroke have a significant reduction in morphometric indices i.e., width of genu, body & splenium, and the AP diameter of the corpus callosum when compared to normal individuals.

## Introduction

The corpus callosum is the link between the two hemispheres of the brain and plays an important role in motor and sensory functions [1,2]. Due to its role in interhemispheric integration, language, intelligence, and creativity are found relatable to the corpus callosum [3,4]. Variations in the dimensions of the corpus callosum are therefore seen in various neurological diseases such as Alzheimer’s and bipolar affective disorder [5-7].

While the dimensions differ based on gender, age, and ethnicity, pathological variations are seen with some diseases such as vascular dementia, leukoaraiosis, stroke, and carotid artery stenosis [8-10]. The corpus callosum is made of five parts namely the body, isthmus, genu, rostrum, and splenium [11]. Genu is anatomically found to be the most anterior and links the medial and lateral frontal lobes, whereas the most posterior aspect is the splenium. The body is the longest and the isthmus is narrow and between the splenium and posterior aspect of the body [12]. T1 weighted magnetic resonance imaging provides the best visualization and helps in assessing the thickness of various segments of the corpus callosum [13,14]. This study was conducted to evaluate corpus callosum dimensions in stroke patients.

## Materials and methods

This was a cross-sectional analytical study conducted on 84 subjects (42 cases and 42 controls) of either gender and age group of 18 years and above where the cases comprised stroke patients with obvious neurologic deficits (sensory/motor/visual); and the control group comprised of patients presenting with complaints of headache, seizures or altered sensorium but with normal MRI and no specific signs and symptoms. The institutional ethics committee, Sri Devaraj Urs Academy of Higher Education and Research, approved the study and granted permission to start the study with approval number DMC/KLR/IEC/405/2022-23 on September 29, 2022. Written informed consent was taken.

Patients found to have large gliotic foci, leukoaraiosis, congenital brain lesions, extra or intra axial brain tumors, previous cranial surgeries, and any disease that could affect corpus callosum thickness such as schizophrenia, seizure disorder, demyelinating diseases, hydrocephalus, or dementia were excluded from the study. MRI of the brain was performed using a 1.5 T 18 channel MR Scanner (Magnetom Avanto, Siemens, Munich, Germany) in patients who met the inclusion criteria. The widths of the genu, body, and splenium and the anterior to posterior diameter were measured using a mid-sagittal view T1 weighted image of the cerebral hemispheres. Rostrum was not included because it is the thinnest portion and it is a beaked segment of the corpus callosum. Sample size was derived using a similar study done by Mohammadi et al., where the mean width ± SD of the rostrum, splenium, and body of the corpus callosum was significantly lower in the stroke patients than in controls (9.84 ± 1.7 vs. 11.20 ± 1.3 mm; 10.32 ± 1.9 vs. 11.98 ± 0.9 mm; and 6.20 ± 1.0 vs. 6.84 ± 0.6 mm respectively) [3]. Assuming alpha error of 5% (95% confidence limit), power of 90%, ratio of cases: control = 1:1. The minimum required sample size to find the difference in mean morphometric values between the two-study groups was taken as 70 subjects (35 cases and 35 controls). The sample size was obtained from the following formula:

The sample size was derived from the following formula:

Sample size (n) = 2*S_P_^2^* [*Z*_1-α/2_* + Z*_1-β_]^2^/μ_d_^2 ^; *S_P_^2^= S_1_^2^+ S_2_^2 ^*/2

where *S_1 _*: Standard deviation in the cases; *S_2 _*: Standard deviation in the controls; μd : Mean difference between the samples; α : Significance level; and 1-β : Power

Data was entered using Microsoft Excel and analyzed using the Statistical Package for Social Science (SPSS) standard version 20.0 (IBM Corp., Armonk, NY). A p-value of <0.05 was considered statistically significant.

## Results

Our study comprised of 84 subjects (42 cases and 42 controls) of which 16 (19.04%) belonged to the age range of 18-40 years, 32 (38.09%) belonged to the age range of 41-60 years and 36 (42.8%) belonged to the age group of >60 years as shown in Table 1. There was no discrepancy between cases and controls or between the age groups as shown via statistical analysis using the Chi square test, with a p-value of 0.26.

**Table 1:** Demographic details of the subjects.

		Cases	Controls	p-value
Age	18-40	4	12	0.260
	41-60	21	11	
	>60	17	19	
Gender	Male	24	23	0.826
	Female	18	19	

The mean widths of the genu, body, and splenium and the anterior to posterior length of the corpus callosum were compared between normal individuals and stroke patients. It was noted to be significantly lesser in cases than in controls as shown in Table 2. The morphometry of genu, body, splenium and the antero-posterior (AP) diameter of the corpus callosum in cases versus controls was noted to be 9.8 ± 1.2 vs. 10.27 ± 0.3 mm, P=0.12; 5.1±0.9 vs. 5.3±0.24 mm, p=0.25; 12.11 ± 9.65 vs. 12.52 ± 13.9 mm, p=0.04 (significant) and 71.22±3.1 vs. 72.32±1.2, p=0.23, respectively as shown in Table 2.

**Table 2:** Morphometric characteristics of the corpus callosum.

	Cases (mm)	Controls (mm)	T value	p-value
Genu	9.8+/-1.2	10.27+/-0.30	1.2	0.12
Body	5.1+/-0.9	5.3+/-0.24	3.4	0.25
Splenium	12.11+/-9.65	12.52+/-13.9	0.9	0.04
Antero-posterior diameter	71.22+/-3.1	72.32+/-1.2	1.8	0.23

However, our study found no statistically significant difference in these indices between male and female subjects (t = 3.55, p = 0.54) as shown in Table 3. 

**Table 3:** Morphometric characteristics of the corpus callosum according to gender.

		Cases	Control	t value	p-value
Male	Genu	9.9+/-2.1	10.54+/-2.7	12.97	0.5
	Body	6.25+/-7.2	6.78+/-7.6	8.2	0.125
	Splenium	11.66+/-4.3	10.58+/-4.5	0.22	0.023
	Antero-posterior diameter	72.09+/-7.6	71.86+/-7.4	14.2	0.11
Female	Genu	9.6+/-1.3	10.04+/-2.8	11.9	0.7
	Body	6.11+/-5.4	6.87+/-1.9	7.23	0.112
	Splenium	10.96+/-2.9	10.2+/-1.5	0.67	0.01
	Antero-posterior diameter	71.9+/-2.7	71.16+/-1.2	12.3	0.8
t = 3.55, p = 0.54; (Not significant)					

**Figure 1:**
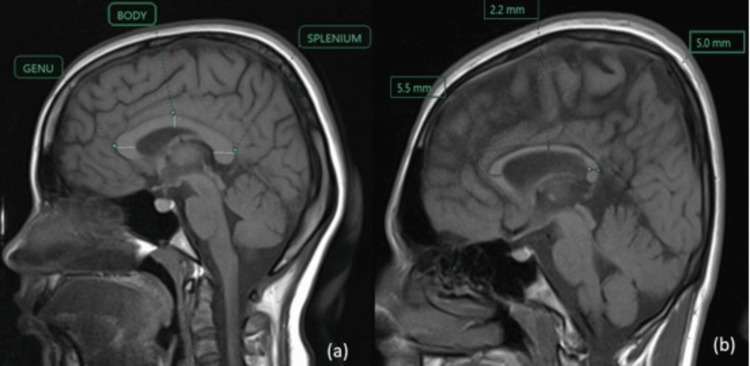
Mid Sagittal T1W magnetic resonance image Images show the measurement of the width of genu, body & splenium at their maximum level in control (a) and reduced width in case (b).

**Figure 2:**
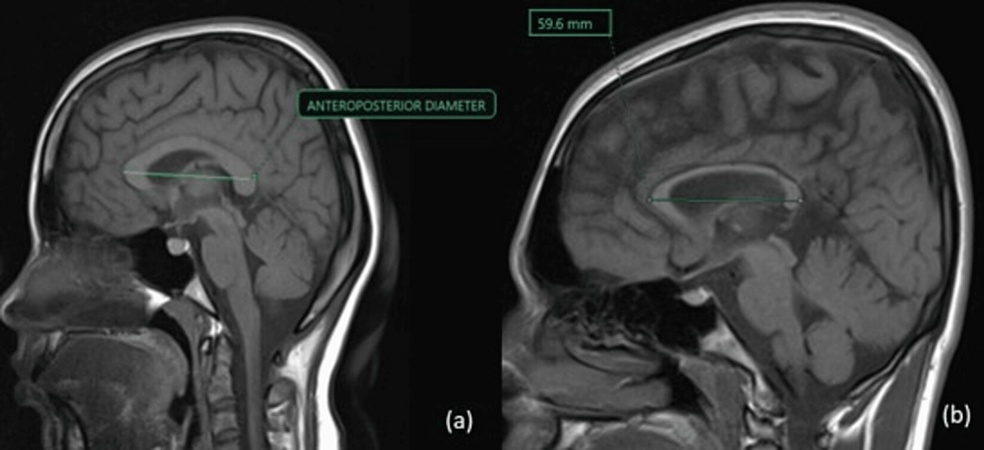
Mid Sagittal T1W magnetic resonance images showing the measurement of the anteroposterior diameter of the corpus callosum Measurement of the anteroposterior diameter of the corpus callosum at its maximum level in control (a) and reduced in case (b).

Our study images are shown in Figures 1-2.

## Discussion

Our study was conducted with the aim of comparing the dimensions of the corpus callosum and making a comparison between normal individuals and stroke patients. As the atrophic changes of the corpus callosum are best identified on MRI, it was the radiological investigation employed for this purpose.

Our analysis revealed that the mean width of the splenium of the corpus callosum was significantly lesser among stroke patients compared with normal individuals. Similarly, other authors like Wu et al. have noted similar results in their study [11]. Wittstock et al. also performed a similar study using T2-weighted MRI and found hyperdense areas in the brain white matter with thinner dimensions [15]. Literature reveals that these changes are chronic in nature and may be due to the demyelination of fibers of the corpus callosum [15]. Even though the morphometry of the corpus callosum varies in the general population, risk factors such as age, and comorbidities such as hypertension and cerebrovascular accidents play a major role. Wu et al. have substantiated this finding through a study they performed using fractional anisotropy whereby they assessed the thicknesses of the genu, body, and splenium of the corpus callosum. They found the dimensions to be much lesser in patients with vascular dementia compared to normal individuals [11].

As our study findings are indicative of obvious changes in morphometric indices in stroke patients, regular follow-up and repeated assessment of these indices with the clinical correlation of neurological sequelae may provide further insight regarding recovery among stroke patients, which was not done in our study but provides scope for future studies in this area. The volume of the corpus callosum was not included in our morphometric indices, the inclusion of which may have provided a better picture for comparison between normal individuals and stroke patients.

## Conclusions

This study showed that patients with stroke have a significant reduction in morphometric indices i.e., width of genu, body & splenium, and the antero-posterior diameter of the corpus callosum when compared to normal individuals. Worldwide, stroke is among the leading causes of long-term motor deficits among elderly individuals due to structural and functional reorganization of specific regions of the brain. Assessment of the changes in corpus callosum morphometry provides a better insight into the correlation in patients post-stroke and may prove crucial in developing better therapeutic interventions for patients.
